# Beyond the Lungs: A Rare Case of Klebsiella pneumoniae Meningitis

**DOI:** 10.7759/cureus.107320

**Published:** 2026-04-19

**Authors:** Sara M Mashal, Rahaf Ghazal, Yousef S Alabrach, Moustafa Hemid, Ahmed Osman

**Affiliations:** 1 Medical Education, Sheikh Khalifa Medical City, Abu Dhabi, ARE; 2 Internal Medicine, Sheikh Khalifa Medical City, Abu Dhabi, ARE

**Keywords:** altered mental status, community-acquired meningitis, klebsiella pneumoniae, spontaneous klebsiella meningitis, tb meningitis

## Abstract

*Klebsiella pneumoniae* is an unusual causative pathogen of meningitis. It is predominantly associated with nosocomial meningitis following traumatic head injury and neurosurgical intervention. Community-acquired *Klebsiella* meningitis, on the other hand, can arise due to prior invasive *Klebsiella pneumoniae* infection. In this article, we present a case of a 48-year-old Asian man presenting with a sudden onset of severe headache, associated with fever and confusion. Early investigations and neuroimaging were suggestive of a central nervous system (CNS) infection, and lumbar puncture confirmed the presence of *Klebsiella pneumoniae*. Targeted antibiotic therapy was initiated, and after a complicated clinical course, the patient improved and was discharged. This case highlights the potential for invasive intracranial disease following recent *Klebsiella pneumoniae* pneumonia and emphasizes the severity and clinical importance of community-acquired *Klebsiella* meningitis.

## Introduction

Bacterial meningitis is an ominous diagnosis due to its severe clinical course and complications, and *Klebsiella* meningitis is considered a rare form of the disease [1]. Recent studies show that it can be divided into two distinct entities: spontaneous (community-acquired) *Klebsiella* meningitis and healthcare-associated (nosocomial) *Klebsiella* meningitis. Nosocomial *Klebsiella* meningitis usually occurs in patients with preexisting intracranial pathologies, surgery, or trauma, while spontaneous *Klebsiella* meningitis occurs in patients with no prior meningeal injury [2,3].

Spontaneous *Klebsiella* meningitis, despite being less common, is associated with a worse outcome than postsurgical *Klebsiella* meningitis, as it is associated with hypervirulent strains of *Klebsiella pneumoniae* that result in significantly higher rates of septic shock and death [2]. *Klebsiella* meningitis shares the common manifestations of other bacterial meningitides, which include fever, signs of meningeal irritation, and altered mental status, as well as headache and seizures [2,3]. Here, we present a case that illustrates the severe nature of spontaneous *Klebsiella* meningitis and highlights the importance of early recognition and treatment.

## Case presentation

A 48-year-old Asian man presented to the emergency department with a sudden onset of severe headache, fever, and multiple episodes of vomiting. He also complained of blurred vision and photophobia. He had severe drowsiness and disorientation to time and place; therefore, clinical information could only be obtained from his relatives. His past medical history was significant for pulmonary tuberculosis (TB) as a child, which was complicated by subsequent bronchiectasis and volume loss of the right lung. He had been hospitalized for *Klebsiella pneumoniae* pneumonia three months prior, which was treated uneventfully with antibiotics. His initial vitals consisted of a temperature of 38.1°C, pulse rate of 150 beats per minute (bpm), respiratory rate of 24/minute, and blood pressure of 162/98 mmHg. The patient’s physical examination was remarkable for severe neck rigidity on passive flexion, but there were no motor or sensory weaknesses. Fundoscopic examination was unremarkable.

Initial investigations revealed a white blood cell (WBC) count of 24.8 × 109/L, a normal hemoglobin, and a severely low platelet count of 35 × 109/L for which lumbar puncture could not be performed (Table 1). The patient was a Jehovah’s Witness; therefore, the offer to transfuse platelets was refused. He was admitted under internal medicine, and due to the severity of his clinical presentation and the suspicion of acute meningitis, he was started on empiric vancomycin, meropenem, acyclovir, and dexamethasone. Moreover, he was given anti-tuberculous medications (ATM) as per his prior history of tuberculosis and significant consciousness impairment.

**Table 1:** Laboratory findings at admission. Abnormal values are highlighted in bold. WBC, white blood cell; AST, aspartate transaminase; ALT, alanine transaminase

Parameter	Result	Reference range
Creatinine	56 mcmol/L	62-106 mcmol/L
Sodium	130 mmol/L	136-145 mmol/L
Potassium	3.3 mmol/L	3.2-5.5 mmol/L
Hemoglobin	138 g/L	131-172 g/L
WBC	24.8 × 10^9^/L	4.5-11.0 × 10^9^/L
Neutrophils	21.9 × 10^9^/L	1.8-7.7 × 10^9^/L
Platelets	35 × 10^9^/L	140-400 × 10^9^/L
CRP	301.00 mg/L	<5.0 mg/L
AST	25 IU/L	<40 IU/L
ALT	111 IU/L	<41 IU/L
Total bilirubin	51.3 mcmol/L	<21.0 mcmol/L

**Figure 1:**
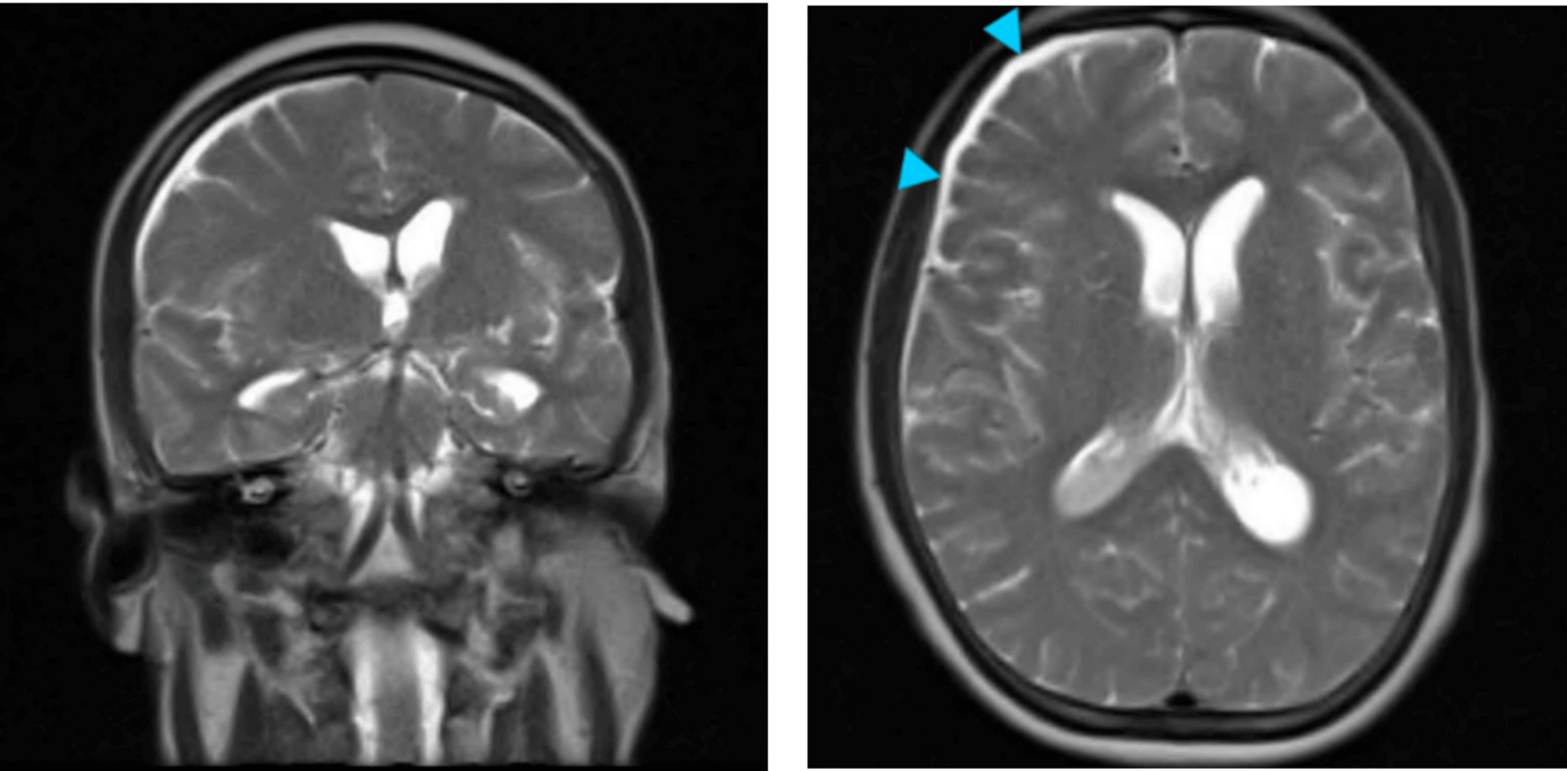
T2 coronal (A) and axial (B) reconstruction of brain MRI demonstrating a mild hydrocephalus of the body and inferior horns of the lateral ventricles. Right frontal subsegmental convexity fluid with CSF signal and mild mass effect is noted (blue arrowheads). CSF: cerebrospinal fluid

**Figure 2:**
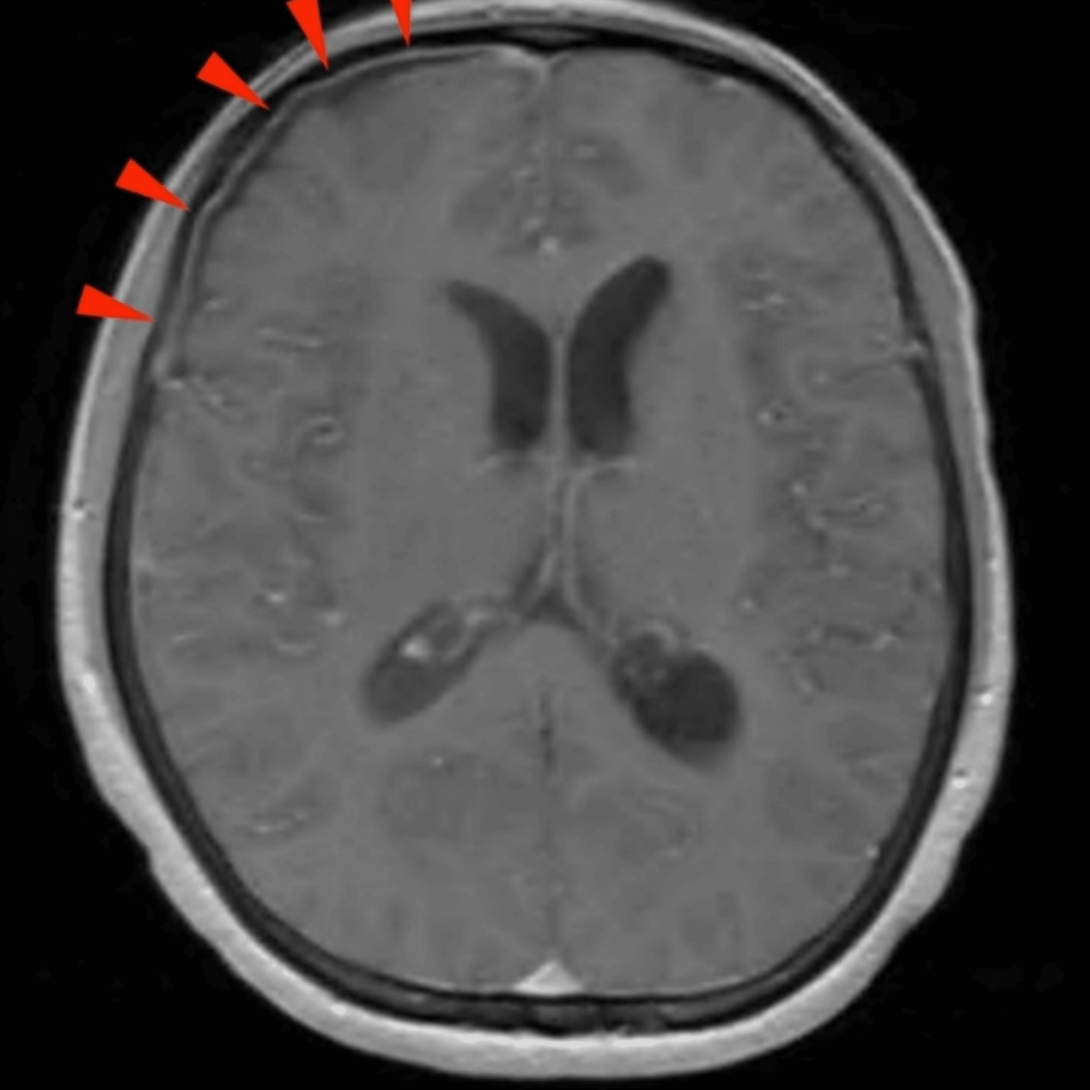
T1 post-contrast axial reconstruction of brain MRI demonstrating localized right frontal outer dural enhancement (red arrowheads) without leptomeningeal involvement.

**Figure 3:**
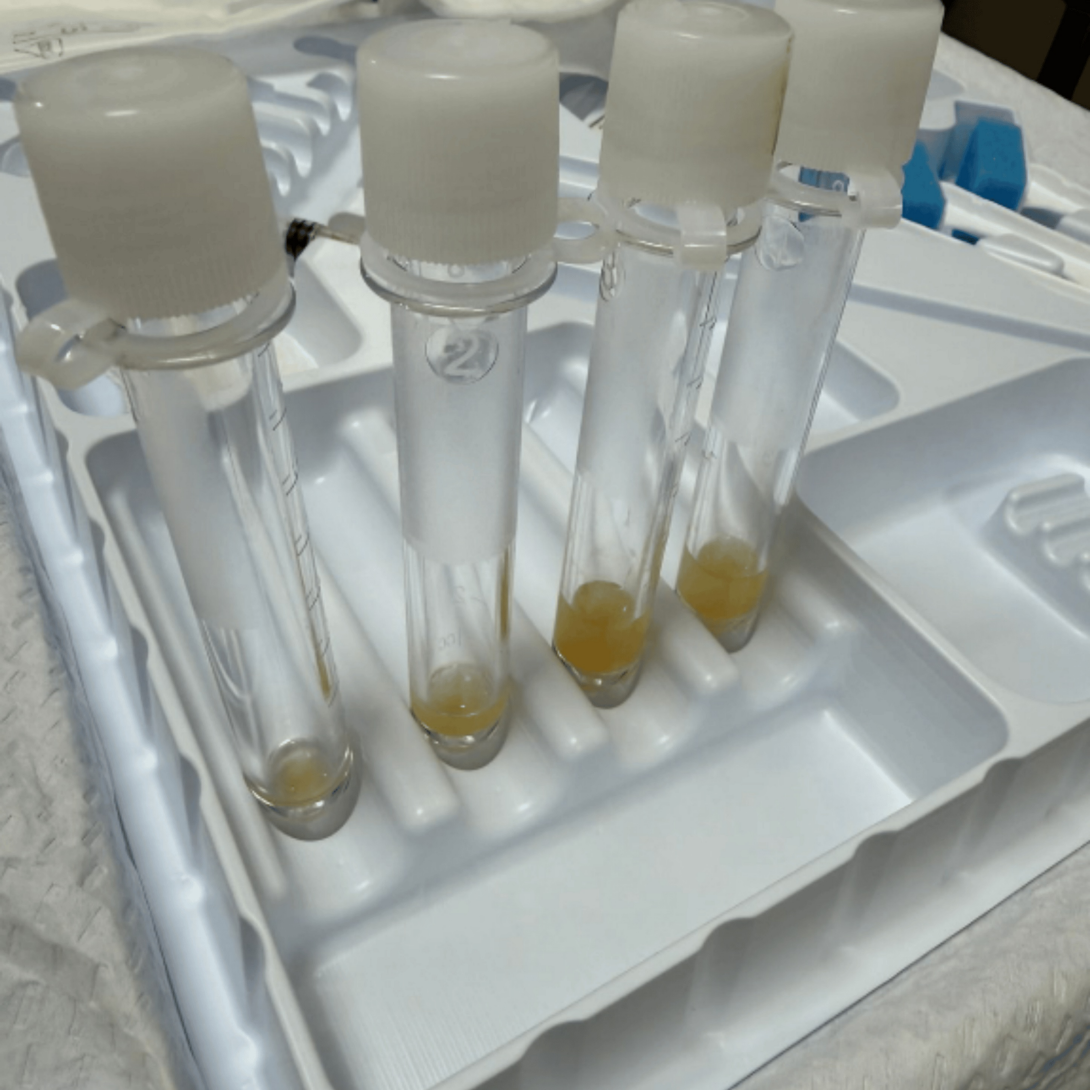
Thick opaque cerebrospinal fluid, consisting mainly of pus.

A brain CT scan was obtained and revealed no acute abnormality. An MRI of the brain was subsequently ordered and was suggestive of meningitis (Figure 1). Over the course of two days, the patient’s platelet count improved; therefore, a lumbar puncture was performed. The gross appearance of the cerebrospinal fluid (CSF) revealed turbid fluid with pus (Figure 2), and his fluid analysis was significant for very low glucose, high protein levels, and a highly neutrophilic infiltrate, a characteristic of bacterial meningitis (Table 2). CSF culture revealed pan-sensitive *Klebsiella pneumoniae *(Figure 3); hence, anti-tuberculous medications and acyclovir were discontinued, and he was switched to ceftriaxone. Blood and sputum cultures were also positive for *Klebsiella pneumoniae*.

**Table 2:** Cerebrospinal fluid analysis.

Test	Result	Reference range
Appearance	Cloudy	Clear, colorless
Nucleated cells	4,886 × 10^6^/L	0-5 ×10^6^/L
Neutrophils	97%	0%
Red blood cells	8,625 × 10^6^/L	0-5 × 10^6^/L
Proteins	4.07 g/L	0.15-0.45 g/L
Glucose	<0.2 mmol/L	>2.1 mmol/L
Lactic acid	13.10 mmol/L	1.10-2.40 mmol/L

Several complications later arose, including a status epilepticus episode, which required the abortive therapy of lorazepam and intensive care for intubation due to a drop in the Glasgow Coma Scale (GCS) score to less than 8. Furthermore, he developed bilateral limb weakness after step-down from the intensive care unit. CT and MRI of the spine were performed and ruled out an acute neurological pathology. He eventually improved with thorough medical management and was discharged on levetiracetam.

## Discussion

The incidence of spontaneous *Klebsiella* meningitis is reported to be as low as 3% of all bacterial meningitis cases in Western countries [1]. Contrastingly, spontaneous *Klebsiella* meningitis appears to be more widespread in the Asia-Pacific rim [2]. Higher gastrointestinal colonization rates of *Klebsiella pneumoniae* have been observed in certain Asian populations, which may contribute to their increased risk [4]. Spontaneous *Klebsiella* meningitis is associated with higher occurrence in middle-aged men with a history of diabetes mellitus, as well as previous invasive *Klebsiella pneumoniae* infection [5]. Preceding extrameningeal infections include pneumonia, pyogenic liver abscesses, endophthalmitis, otitis media, and urinary tract infections [6]. Other risk factors implicated are alcoholism and liver cirrhosis [2,3].

Our patient was an Asian man with no prior neurosurgical history, diabetes, or liver cirrhosis; however, his recent history of hospitalization for *Klebsiella pneumoniae* pneumonia likely facilitated the progression into a meningeal infection. Since *Klebsiella pneumoniae* is associated with the development of pyogenic liver abscess, abdominal ultrasound can be performed to assess for extrameningeal manifestations [6]. In our case, the patient’s ultrasound showed gallbladder polyps but no hepatic cysts or abscesses.

The prompt initiation of antibiotic treatment is essential, as severe complications such as sudden death due to sepsis, disseminated intravascular coagulation (DIC), cerebral edema, or intractable seizures can occur [6,7]. Overall, community-acquired *Klebsiella* meningitis tends to be responsive to beta-lactam antibiotics, particularly third-generation cephalosporins [2]. In contrast, nosocomial *Klebsiella* meningitis displays higher rates of extended-spectrum beta-lactamase production; hence, it is associated with higher rates of antibiotic resistance [2]. Furthermore, the patient’s history of pulmonary tuberculosis (TB), along with his deteriorating cognitive condition, made TB meningitis an important differential diagnosis, as altered mental status is a specific manifestation of TB meningitis [8]. Accordingly, anti-tuberculous medications were initiated in lieu of our inability to obtain CSF analysis beforehand. Once a lumbar puncture could be performed and *Klebsiella* meningitis was confirmed, anti-tuberculous medications were promptly stopped. Empiric coverage for TB was justifiable, as the morbidity and mortality of TB meningitis are significantly high [9].

Our patient developed decreased consciousness and status epilepticus requiring intensive care support, reflecting the aggressive nature of the disease. Nevertheless, early empiric therapy, followed by culture-directed antibiotics, resulted in clinical recovery.

## Conclusions

Community-acquired *Klebsiella pneumoniae* meningitis is a rare but potentially life-threatening infection that may occur even in the absence of traditional risk factors such as diabetes mellitus or hepatic abscesses. Compared with nosocomial disease, community-acquired infection may follow a more severe course and develop after a recent pulmonary infection. The early recognition and prompt initiation of empiric broad-spectrum antimicrobial therapy are essential, especially when diagnostic procedures such as lumbar puncture are delayed.
